# Drip Fertigation Enhances the Responses of Grain Yield and Quality to Nitrogen Topdressing Rate in Irrigated Winter Wheat in North China

**DOI:** 10.3390/plants13111439

**Published:** 2024-05-22

**Authors:** Jin Tong, Yulei Xiong, Yu Lu, Wen Li, Wen Lin, Jianfu Xue, Min Sun, Yuechao Wang, Zhiqiang Gao

**Affiliations:** College of Agriculture, Shanxi Agricultural University, Jinzhong 030801, China; tongjin0720@163.com (J.T.); xiongyulei1001@163.com (Y.X.); luyu0607ly@163.com (Y.L.); liwen001216@163.com (W.L.); slwrdewy@163.com (W.L.); fudange95@163.com (J.X.); sm_sunmin@126.com (M.S.)

**Keywords:** drip fertigation, nitrogen topdressing rate, wheat yield, grain quality, radiation use efficiency

## Abstract

Conventional water and nitrogen (N) management practice in north China, comprising flood irrigation and N fertilizer broadcast (FB), limits sustainable wheat production. Drip fertigation (DF) has been widely adopted in wheat production in recent years and has effectively improved yields. However, the responses of the yield and quality to the N topdressing rate (NTR) under DF are still unclear. This study determined the responses of the wheat yield and quality to NTR under DF, as well as assessing whether DF could synergistically increase the yield and quality. A field experiment was conducted in north China for two seasons (2021–2023) using a split-plot design with three replicates. The main plot used the management practice (FB and DF) and the sub-plot had N treatment (no N applied, and NTRs of 0, 40, 80, 120, and 160 kg ha^−1^ with 150 kg N ha^−1^ as basal fertilizer, denoted as N0, T0, T40, T80, T120, and T160, respectively). Our results showed that high and saturated wheat yields (12.08 and 11.46 t ha^−1^) were obtained under DF at T80, and the highest yields were produced at T160 (11.71 and 11.30 t ha^−1^) under FB. Compared with FB, the greatest yield increase of 10.4–12.6% was achieved at T80 under DF. A higher spike number due to the increased effective stem percentage and a greater grain weight because of enhanced post-anthesis biomass production (BP_post_) explained the improved yield under DF. The enhanced post-anthesis radiation use efficiency (RUE) led to the greater BP_post_ under DF. The enhanced specific leaf N, antioxidant capacity, and stomatal conductance under DF explained the higher light-saturated photosynthesis rate of flag leaves, which partly led to the increased post-anthesis RUE. NTR higher than 80 kg ha^−1^ did not enhance the yield, but it significantly improved the gliadin and glutelin contents, thereby leading to a higher total protein content, better gluten characteristics, and superior processing quality. Therefore, drip fertigation is a practical strategy for increasing both yield and quality with reduced water input and appropriate N input in irrigated winter wheat in north China. Applying 80 kg ha^−1^ of NTR under drip irrigation produces a high yield, but further gain in grain quality needs a higher NTR.

## 1. Introduction

Wheat (*Triticum aestivum* L.) plays a vital role in food security in China and worldwide, and the area planted with wheat throughout the world and in China specifically comprises approximately 17% and 24% of the total area planted with cereal crops, respectively [1]. In north China, the total winter wheat planting area and yield in Henan, Shandong, Hebei, Shaanxi, and Shanxi provinces account for 57% and 62% of the total in China, respectively [2]. In this region, precipitation during the growing season of winter wheat could only provide 25–30% of the water requirements [3,4]. Thus, it is important to apply supplementary irrigation to ensure that the water supply is sufficient for high and stable winter wheat production. The dominant irrigation method used in wheat production in north China is conventional flood irrigation. However, the large amounts of water consumed in the conventional irrigation method lead to the inefficient utilization of limited water resources [5], which is not conducive to sustainable food production [6,7].

The application of nitrogen (N) fertilizer is also critical for the growth of wheat plants and yield formation. The leaves are fewer in number and/or smaller when the N supply is insufficient, which inhibits the canopy radiation capture and photosynthesis capacity, thereby reducing the crop grain yield and quality [8,9,10]. Appropriate N management determines increases in the wheat grain yield and quality improvements [11]. Under the conventional flood irrigation method, N fertilizer is usually broadcast on the soil surface or buried in the topsoil. After irrigation with a large amount of water, a considerable proportion of the N infiltrates into the deep layer soil, where N fertilizer cannot be absorbed sufficiently by the limited roots [12,13,14]. Water and N fertilizer management practices are characterized by flood irrigation and N fertilizer broadcast (FB), which limits the efficient use of water and N fertilizer, and it also leads to a high risk of nitrate leaching [15,16]. Therefore, it is necessary to improve water and N management practices to increase crop productivity and the efficiency of resource use, as well as to reduce negative ecological impacts.

An effective management solution is drip fertigation (DF), which can supply water and fertilizer simultaneously to the crop root zone by controlling a drip irrigation system in a timely and precise manner according to the water and nutrient requirements of crops [17,18,19]. DF has been widely used in vegetable and fruit production [20], and it has recently become increasingly applied in cereal crop production in areas with water scarcity [12,21,22]. Numerous studies have shown that DF can achieve the combined goals of reducing water usage, increasing N use efficiency, and obtaining high yields. DF can significantly increase crop yields by 10–22% compared with traditional irrigation and fertilization practices [23,24,25]. Lu et al. [14] found that compared with FB, DF increased the wheat yield, water productivity, N fertilizer use efficiency, and net income by 43–56%, 44–54%, 47–111%, and 88–100% compared with FB, respectively. Li et al. [26] analyzed 1033 publications and concluded that compared with FB, DF could increase the crop yield, water productivity, and N fertilizer use efficiency by 12%, 26%, and 34%, respectively. These studies mostly focused on the crop yield, water evapotranspiration and productivity, and N uptake and utilization, whereas few considered biomass production, translocation, and partitioning, which primarily determine the crop yield performance.

Crop yield is dependent on both biomass production and partitioning (i.e., harvest index). The grain yields from modern wheat varieties are mainly improved by increasing the accumulation of biomass, and it is difficult to further increase the harvest index by genetic improvement or enhanced cultivation [27,28]. According to crop growth analysis, two physiological traits comprising the canopy radiation interception and radiation use efficiency (RUE) have key roles in determining the crop biomass [29,30]. The speed of canopy development and closure, as well as the architecture of the canopy, determine the interception of radiation. In particular, the leaf area index (LAI) plays an important role in determining the capacity of radiation capture [31,32]. The RUE reflects the efficiency of converting the radiation interception into biomass, regarding the fundamental bottleneck that hinders improvements in wheat yields [33]. Moreover, RUE is sensitive to the leaf N conditions, especially the N content per unit leaf area (SLN, g N m^2^) [34,35,36]. Biomass increases can be achieved by improving one or both parameters. However, little information is available about the responses of canopy radiation interception and RUE to different water and N management practices.

Due to plant absorption and N losses by nitrate leaching and ammonia volatilization during the long winter dormancy period, the residual basal N fertilizer typically cannot meet the requirements for rapid plant growth and development. Therefore, the application of N fertilizer topdressing in the spring is widely adopted in irrigated winter wheat production. Wen et al. [37] showed that an appropriate N topdressing rate (NTR) can increase the effective stem percentage, spike number, and grain weight, and thus the grain yield. Zhao et al. [38] and Ma et al. [39] showed that the flag leaf chlorophyll content and net photosynthesis rate, total biomass, harvest index, and yield increased as the NTR increased. In addition, these studies showed that the protein content, wet gluten content, dough development, and stability time improved as NTR increased [40]. Guo et al. [41] found that the protein content, wet gluten content, and dough stability time increased continuously as the NTR increased from 0 to 180 kg ha^−1^, although the peak yield was achieved at 150 kg ha^−1^. These previous studies were all conducted under FB, whereas little information is available about how different NTRs under DF might affect the grain yield and quality. Thus, it is necessary to obtain this information in order to optimize N management strategies under DF to facilitate yield and quality improvements.

Therefore, in the present study, based on a field experiment conducted for two years, we investigated the effects of different management practices (FB and DF) and NTR on the radiation interception and RUE, biomass, yield, and quality of winter wheat in the southern Shanxi province of north China. The purposes of this study were (1) to identify the responses of the yield and quality to NTR under different management practices and (2) to clarify the underlying physiological mechanisms related to the responses according to radiation interception and RUE. We hypothesized that DF would improve both the yield and quality by strengthening their responses to NRT, and that increasing RUE would explain the more sensitive response of the yield under DF.

## 2. Results

### 2.1. Yield and Yield-Related Attributes

**Figure 1:**
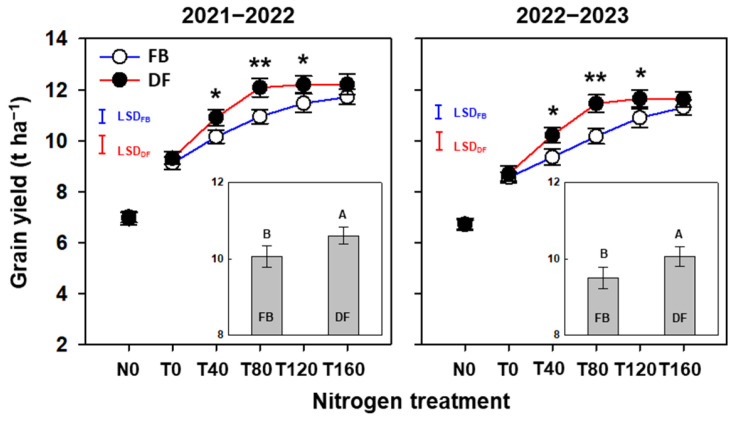
Grain yield of winter wheat in the 2021–2022 and 2022–2023 growing seasons. The different uppercase letters indicate there is a significant difference between FB and DF averaged across all nitrogen treatments according to the least significant difference test (LSD, α = 0.05). Here, * and ** indicate there is a significant difference between FB and DF according to Student’s *t* test at *α* = 0.05 and 0.01, respectively. The blue and red vertical bars represent the least significant differences under FB and DF (*p* < 0.05), respectively.

Averaged across N treatments, compared with FB, the grain yield increased significantly by 5.4–5.9% under DF (Figure 1). Under FB, the yield increased significantly and continuously as NTR increased from 0 to 160 kg ha^−1^. Under DF, the yields increased significantly and continuously as NTR increased from 0 to 80 kg ha^−1^, but no significant differences in the yields were found between T80, T120, and T160. There were no significant differences in the yields between management practices at N0, T0, or T160 in both seasons. Compared with FB, the yields at T40, T80, and T120 under DF increased significantly by 7.4–9.2%, 10.4–12.6%, and 6.5–6.9%, respectively. In addition, the yield at T80 under DF was significantly higher and almost equivalent to the yields at T120 and T160 under FB.

The spike number, grains per spike, and grain weight were all significantly affected by management practice, N treatment, and their interactions, except for grains per spike, which was not significantly affected by the management practice (Table 1). Averaged across N treatments, compared with FB, the spike number and grain weight increased significantly by 5.0–5.4% and 4.0–4.3% under DF, respectively, thereby resulting in higher yields under DF. As NTR increased, the spike number and grains per spike tended to increase, but the grain weight decreased. The spike number and grains per spike did not significantly increase when NTR increased from 80 to 160 kg ha^−1^ under DF, but significant increases were observed under FB. Similar to the yield performance, the spike number at T80 under DF was not lower than that at T160 under FB.

Rather than the maximum stem number, the higher productive stem percentage (by 6.3–6.8%) could explain the increased spike number under DF (Table 1). There were no significant differences in LAI at anthesis under the two management practices in both seasons. Under both management practices, LAI at anthesis increased significantly as NTR increased from 0 to 80 kg ha^−1^, but no significant increase was found as NTR increased from 80 to 160 kg ha^−1^.

### 2.2. Biomass Accumulation

**Figure 2:**
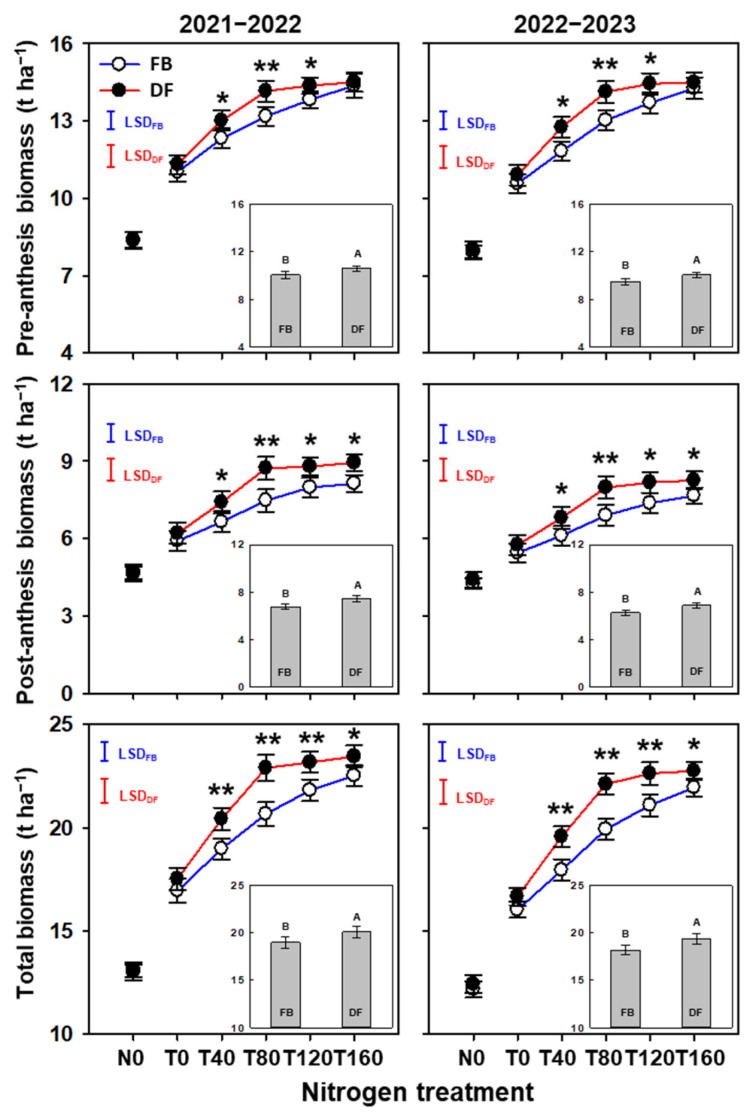
Pre-anthesis, post-anthesis, and total biomass in the 2021–2022 and 2022–2023 growing seasons. The different uppercase letters indicate there is a significant difference between FB and DF averaged across all nitrogen treatments according to the least significant difference test (LSD, α = 0.05). Here, * and ** indicate there are significant differences between FB and DF according to Student’s *t* test at *α* = 0.05 and 0.01, respectively. The blue and red vertical bars represent least significant differences under FB and DF (α = 0.05), respectively.

Averaged across N treatments, compared with FB, the total biomass increased significantly by 5.8–6.5% under DF (Figure 2). The increase in the total biomass was due to the simultaneous improvements in BP_pre_ and BP_post_ (by 3.6–4.8% and 9.6–9.8%, respectively). Similar to the trend in the yield, BP_pre_, BP_post_, and the total biomass all increased as NTR increased from 0 to 160 kg ha^−1^ under FB, but significant increases were only found from 0 to 80 kg ha^−1^ and the biomass was unchanged from 80 to 160 kg ha^−1^ under DF.

Compared with FB, BP_pre_ improved significantly at T40, T80, and T120 by 5.6–7.9%, 7.4–8.5%, and 4.0–5.4% under DF, respectively (Figure 2), where the corresponding percentages for BP_post_ were 11.3–11.4%, 15.7–16.7%, and 10.1–10.9%, and the corresponding percentages for the total biomass were 7.6–9.1%, 10.8–11.0%, and 6.2–7.3%. In particular, BP_post_ was 7.7–9.9% higher at T160 under DF compared with FB.

### 2.3. NSC Accumulation and Biomass Translocation and Partitioning

The NSC accumulation at anthesis, B_trans_, NSC output rate, and the contribution rate of B_trans_ to the yield were all affected significantly by the management practice, N treatment, and their interaction, except that the management practice had no significant effect on NSC accumulation or B_trans_ (Table 2). Averaged across N treatments, compared with FB, the NSC output rate and contribution rate of B_trans_ to the yield were 10.1–11.1% and 9.1–13.7% lower under DF, respectively. HI was not significantly affected by the management practice. Under both management practices, small differences in HI were observed among all NTRs (0–160 kg ha^−1^) in both seasons.

**Figure 3:**
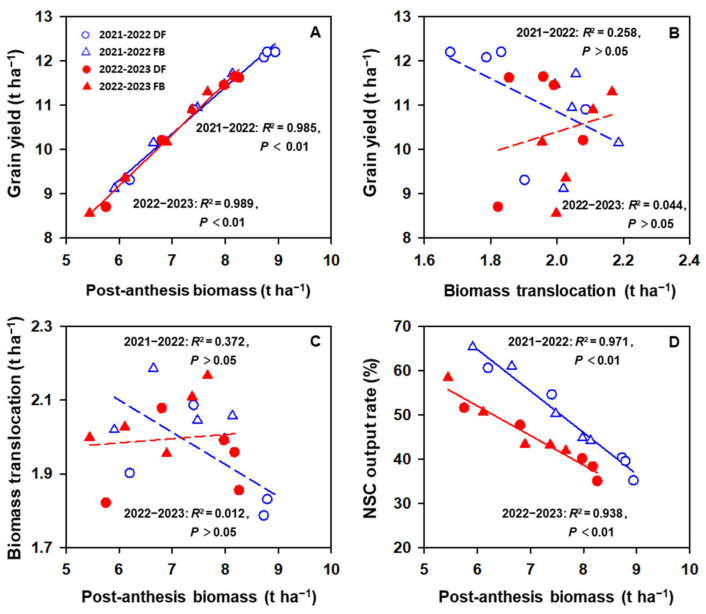
Relationship between yield and post-anthesis biomass (**A**) or biomass translocation (**B**), and between biomass translocation (**C**) or NSC output rate (**D**) and post-anthesis biomass.

There was no significant relationship between the grain yield and B_trans_ (Figure 3B, *p* > 0.05) in either year, whereas the grain yield was significantly and positively correlated with BP_post_ (Figure 3A, *p* < 0.01). BP_post_ was not correlated with B_trans_ (Figure 3C, *p* > 0.05). BP_post_ was significantly and negatively correlated with the NSC output rate (Figure 3D, *p* < 0.01).

### 2.4. PAR Interception and Use Efficiency

**Figure 4:**
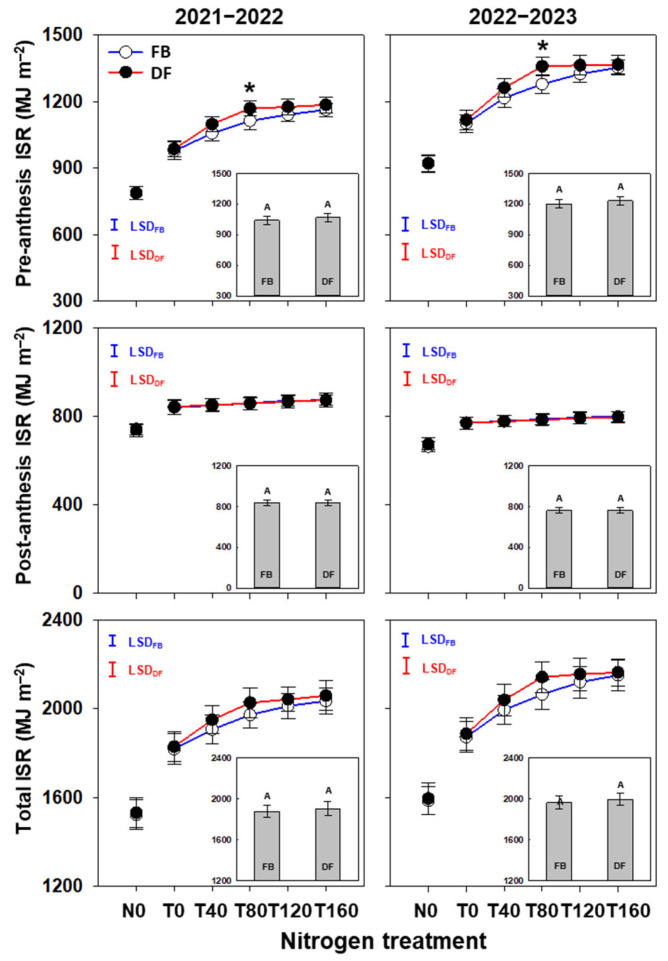
Pre-anthesis, post-anthesis, and total ISR in the 2021–2022 and 2022–2023 growing seasons. The different uppercase letters indicate there is a significant difference between FB and DF averaged across all nitrogen treatments according to the least significant difference test (LSD, α = 0.05). Here, * indicates that there is significant difference between FB and DF according to Student’s *t* test at *α* = 0.05. The blue and red vertical bars represent least significant differences under FB and DF (α = 0.05), respectively.

Averaged across N treatments, the pre-anthesis, post-anthesis, and total ISR did not differ significantly between the two management practices (Figure 4). The pre-anthesis and total ISR increased significantly as NTR increased from 0 to 80 kg ha^−1^ under both management practices. No significant differences were found under DF as NTR increased from 80 to 160 kg ha^−1^, but the pre-anthesis ISR improved significantly at T160 compared at T80 under FB. Moreover, the pre-anthesis ISR was 5.1–6.3% higher at T80 under DF than FB.

**Figure 5:**
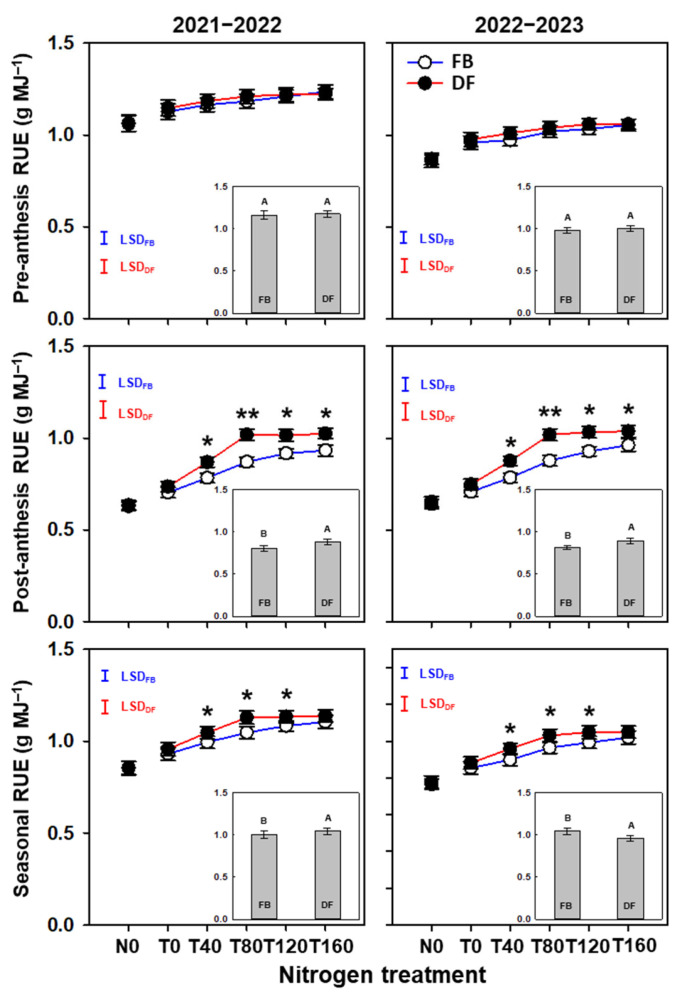
Pre-anthesis, post-anthesis, and total RUE in the 2021–2022 and 2022–2023 growing seasons. The different uppercase letters indicate there is a significant difference between FB and DF averaged across all nitrogen treatments according to the least significant difference test (LSD, α = 0.05). Here, * and ** indicates there is significant difference between FB and DF according to Student’s *t* test at *α* = 0.05 and 0.01, respectively. The blue and red vertical bars represent least significant differences under FB and DF (α = 0.05), respectively.

Averaged across N treatments, the pre-anthesis RUE did not differ significantly between the two management practices, but the post-anthesis and seasonal RUE were 9.5–9.6% and 4.1–4.6% higher under DF than FB, respectively (Figure 5). As NTR increased, the pre-anthesis RUE tended to increase slightly under both management practices in both years. Under FB, the post-anthesis and seasonal RUE tended to increase continuously as NTR increased from 0 to 160 kg ha^−1^. However, the post-anthesis and seasonal RUE did not differ significantly among T80, T120, and T160 under DF. Compared with FB, the post-anthesis RUE values were 11.0–11.6%, 16.0–16.9%, 10.7–11.3%, and 7.8–9.7% higher at T40, T80, T120, and T160 under DF, respectively. Compared with FB, the seasonal RUE values were 5.2–6.7%, 6.9–7.8%, and 4.7–5.6% higher at T40, T80, and T120 under DF, respectively.

### 2.5. Photosynthetic Characteristics of Flag Leaves after Anthesis

**Figure 6:**
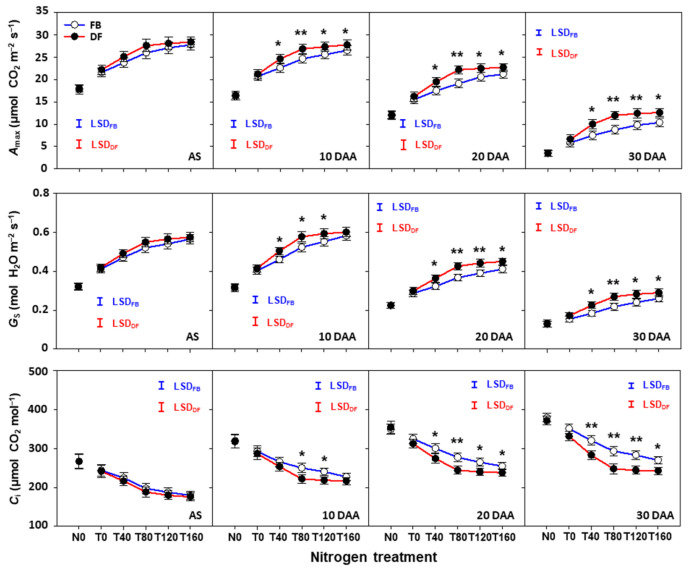
Light-saturated photosynthetic rate (*A*_max_), stomatal conductance (*G*_s_), and intercellular CO_2_ concentration (*C*_i_) of flag leaves after anthesis in the 2021–2022 growing season. Here, * and ** indicate that there is significant difference between FB and DF according to Student’s *t* test at α = 0.05 and 0.01, respectively. The blue and red vertical bars represent the least significant differences under FB and DF (α = 0.05), respectively.

**Figure 7:**
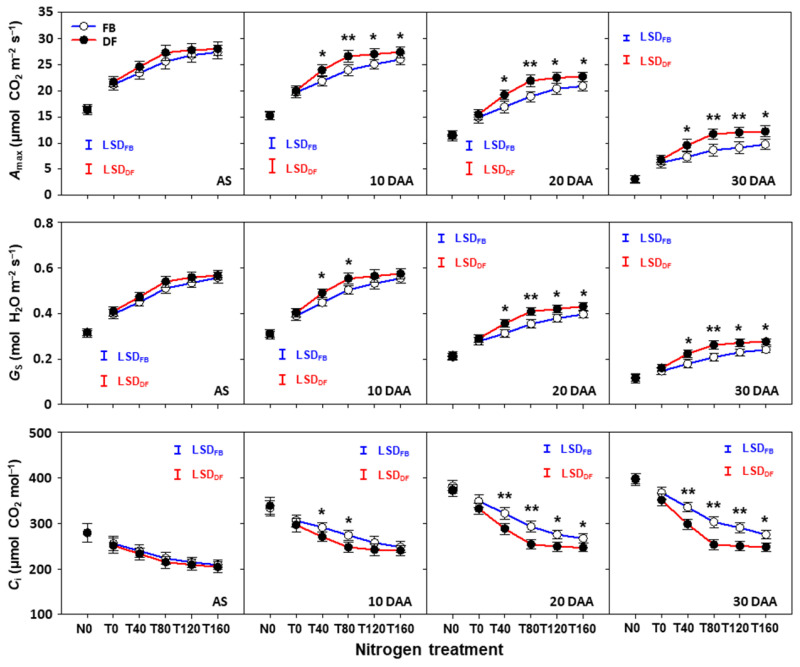
Light-saturated photosynthetic rate (*A*_max_), stomatal conductance (*G*_s_), and intercellular CO_2_ concentration (*C*_i_) of flag leaves after anthesis in the 2022–2023 growing season. Here, * and ** indicate that there is significant difference between FB and DF according to Student’s *t* test at α = 0.05 and 0.01, respectively. The blue and red vertical bars represent least significant differences under FB and DF (α = 0.05), respectively.

The *A*_max_ and *G*_s_ of flag leaves after anthesis decreased during the grain filling period (from 10 to 30 DAA) under all N treatments, management practices, and seasons, whereas *C*_i_ increased (Figure 6 and Figure 7). *A*_max_ and *G*_s_ increased as NTR increased from 0 to 160 kg ha^−1^ under FB, but there was no obvious change from 80 to 160 kg ha^−1^ under DF. However, *C*_i_ decreased as NTR increased from 0 to 160 kg ha^−1^ under FB, although obvious decreases were only found from 0 to 80 kg ha^−1^ under DF. There was no significant difference in *A*_max_ and *G*_s_ at anthesis between management practices under any N treatment in either season. The difference percentages between management practices in *A*_max_ and *G*_s_ became larger over time. At 10, 20, and 30 DAA, the *A*_max_ values under DF were 4.7–11.0%, 7.1–16.2%, and 21.5–36.7% higher at NTRs of 40–160 kg ha^−1^ than for FB, respectively. For *G*_s_, the corresponding increase percentages were 3.1–10.4%, 8.8–16.0%, and 11.4–25.6%. However, for *C*_i_, corresponding decrease percentages were 3.0–9.6%, 6.5–13.3%, and 9.9–16.4%.

### 2.6. SLN Contents of Flag Leaf after Anthesis

**Figure 8:**
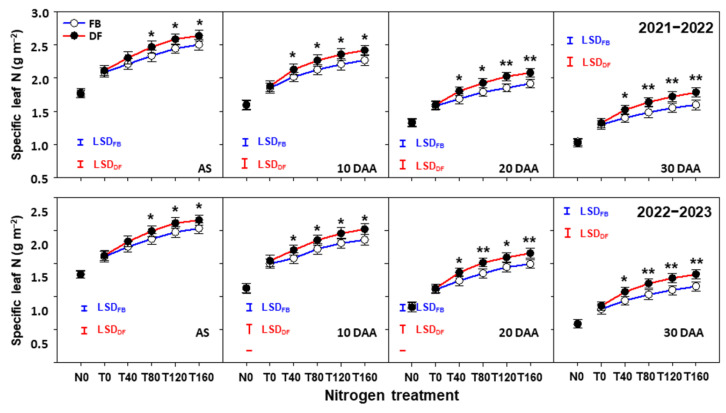
Specific leaf N (SLN) of flag leaves after anthesis in the 2021–2022 and 2022–2023 growing seasons. Here, * and ** indicate that there is significant difference between FB and DF according to Student’s *t* test at α = 0.05 and 0.01, respectively. The blue and red vertical bars represent least significant differences under FB and DF (α = 0.05), respectively.

In the same way as *A*_max_, the SLN contents of flag leaves after anthesis decreased over time under all N treatments, management practices, and seasons (Figure 8). The continuous improvements in the SLN contents were observed under both practices as NTR increased. The difference percentages in SLN also became larger over time. The SLN contents were 4.9–5.8%, 5.5–6.8%, 6.9–9.4%, and 8.8–11.7% higher at NTR of 40–160 kg ha^−1^ at anthesis, and 10, 20, and 30 DAA under DF than FB, respectively.

### 2.7. CAT and SOD Activities and MDA Contents of Flag Leaves after Anthesis

The CAT and SOD activities in the flag leaves decreased over time under both management practices in both seasons (Figures S1 and S2). At anthesis, there were no differences in the CAT or SOD activities between the two management practices under any N treatments. As time went by, the DF demonstrated larger and larger advantages in the CAT and SOD activities. At 30 DAA, the CAT and SOD activities were 10.7–15.3% and 7.5–13.0% higher at NTRs of 40–160 kg ha^−1^ under DF than FB, respectively.

The MDA contents of the flag leaves increased under both management practices in the two seasons (Figures S1 and S2). No significant difference was found in the MDA contents between management practices either at anthesis or 10 DAA under any N treatment or in any season. The MDA contents were 4.8–6.4% and 5.3–8.4% higher at 20 and 30 DAA at NTRs of 40–160 kg ha^−1^ under DF than FB, respectively.

### 2.8. Grain Protein Content and Protein Components

In both seasons, the gliadin, glutenin, and total protein content were significantly affected by the management practice, N treatment, and their interaction, but the albumin plus globulin was not affected by any factor (Table 3). Averaged across N treatments, the gliadin, glutenin, and total protein contents were 5.4–6.1%, 4.8–5.0%, and 3.9–4.1% higher under DF than FB, respectively. As NTR increased from 0 to 160 kg ha^−1^, the gliadin, glutenin, and total protein contents all tended to increase under both management practices and in both seasons. Under FB, no significant improvements were observed as NTR increased from 80 to 160 kg ha^−1^. By contrast, the gliadin, glutenin, and total protein contents were 9.6–10.9%, 7.9–8.5%, and 6.6–7.7% higher at T160 than those at T80 under DF, respectively. Furthermore, the gliadin, glutenin, and total protein contents were 7.4–12.6%, 6.8–10.4%, and 5.4–8.8% higher at NTRs of 120 and 160 kg ha^−1^ under DF than FB, respectively.

### 2.9. Grain Processing Quality

**Figure 9:**
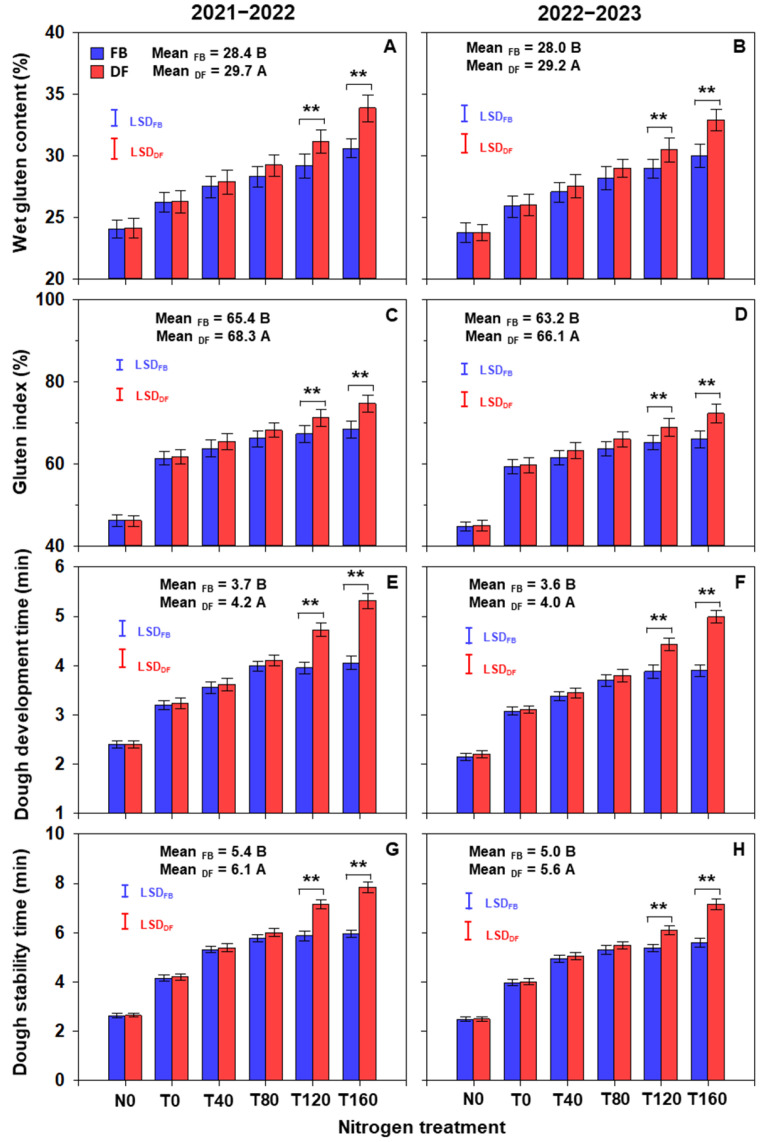
The wet gluten content (**A**,**B**), gluten index (**C**,**D**), dough development time (**E**,**F**) and stability time (**G**,**H**) at maturity in the 2021–2022 and 2022–2023 growing seasons. Means followed by different uppercase letters are significantly different according to the least significant difference test (LSD, α = 0.05) between two management patterns. Here, ** indicates that there is significant difference between FB and DF according to Student’s *t* test at α = 0.05. The blue and red vertical bars represent least significant differences under FB and DF (α = 0.05), respectively.

Averaged across N treatments, the wet gluten content and gluten index were 4.1–4.7% and 4.4–4.5% higher under DF than FB, respectively (Figure 9A–D). As NTR increased, the wet gluten content and gluten index increased continuously under both practices, but the increasing trends were stronger under DF. Under FB, the wet gluten content and gluten index were 15.8–16.6% and 11.1–11.4% higher at T160 than T0, respectively, but the corresponding percentages under DF were 26.6–28.9% and 21.0–21.1%. At NTRs of 120 and 160 kg ha^−1^, the wet gluten content and gluten index were 5.3–10.7% and 5.7–9.5% higher under DF than those under FB, respectively.

Averaged across N treatments, the dough development time and stability time were 10.3–12.0% and 10.3–13.0% higher under DF than FB, respectively (Figure 9E–H). As NTR increased, both the development time and stability time increased continuously under DF, whereas the values did not increase when NTR exceeded 80 kg ha^−1^ under FB. Under DF, the dough development time and stability time were 29.5–31.3% and 30.2–30.6% higher at T160 than T80, respectively. At NTRs of 120 and 160 kg ha^−1^, the dough development time and stability time were 14.4–31.1% and 13.7–31.7% higher under DF than FB, respectively.

## 3. Discussion

Nitrogen fertilizer topdressing is essential for improving wheat productivity and grain quality. Enhancing the responses of yield and quality to NTR may be one of the most effective and meaningful ways to ensure food security without very high nitrogen fertilizer input. In the present study, the average grain yield was 5.4–5.9% higher under DF compared with FB, and similar results were obtained in previous studies [14,24,26]. Importantly, our results showed that the response of the wheat yield was more sensitive to NTR under DF than FB. High yield was achieved at T80 under DF, but no obvious peak in the yield was observed as NTR increased from 0 to 160 kg ha^−1^ under FB. In addition, N topdressing at 80 kg ha^−1^ under DF produced an equivalent yield to that at N topdressing at 160 kg ha^−1^ under FB in both seasons (12.08 vs. 11.71 t ha^−1^ in 2021−2022, and 11.46 vs. 11.30 t ha^−1^ in 2022−2023). These results suggest that DF can strengthen the response of the grain yield to NTR to obtain high yields without requiring high N topdressing inputs to facilitate more sustainable agriculture.

Overall, the yield improvements under DF were attributed to the increases in the number of spikes per 1 m^2^ and grain weight. The improvement in the number of spikes per 1 m^−2^ under DF was due to the increase in the productive stem percentage rather than the maximum stem number, which was mainly due to conducting N topdressing in the jointing stage when the stem number had already reached the maximum value, before declining due to the fierce competition for nutrients, water, and space. The N supply plays a critical role in stem development or death [32,42,43]. It has been widely reported that DF can provide a higher available N concentration in the topsoil layer for absorption by the roots and subsequent utilization by the shoots [17,44,45]. The greater available N supply under DF could allow a higher proportion of stems to develop into productive spikes. In terms of grain production, both BP_post_ and B_trans_ determine the grain weight, and thus the grain yield [46,47]. We found that B_trans_ did not differ under DF and FB, but BP_post_ was 9.6–9.8% higher under DF. Thus, we mainly attributed the higher grain weight and yield to the higher BP_post_ values in both seasons. B_trans_ depends on the total NSC accumulation at anthesis and the NSC output rate. We found no significant increase in the total NSC accumulation under DF but a significant decrease in the NSC output rate. In addition, the NSC output rate was significantly and negatively correlated with BP_post_ in each year, as also shown in previous studies [48,49,50]. These results suggest that the increase in BP_post_ under DF was important for increasing the grain yield, but it also limited the translocation of NSC reserves into the filling grain and restricted further yield improvements. It is desirable to develop management practices or breed new cultivars that will break the negative relationship between BP_post_ and the NSC output to further enhance wheat yields.

The crop yield is determined by the biomass and HI. However, biomass increases have contributed more to yield improvements than HI in recent decades [27,28,51]. We found that DF increased both the pre-anthesis and post-anthesis biomass to achieve a higher total biomass, but HI was not affected. The increase in the total biomass under DF was mainly dependent on BP_post_ (9.6–9.8%) rather than BP_pre_ (3.6–4.8%). The notably larger increase in BP_post_ under DF was attributed to the higher RUE rather than ISR. When N topdressing was conducted, the post-anthesis RUE was 7.8–16.9% higher under DF than FB. Furthermore, the post-anthesis RUE under DF at T80 was also significantly higher than or equivalent to that at T160, which produced the highest RUE under FB. These results suggest that the more sensitive response of the post-anthesis RUE mainly contributed to the greater response of the yield to NTR under DF.

Flag leaf assimilates make the most important contribution to the grain yield [52,53,54], and thus the photosynthetic capacity of flag leaves is generally used as an indicator of canopy productivity [55,56]. We found that DF significantly improved *A*_max_, which was the result of the greater *G*_s_ of flag leaves at NTRs of 40–160 kg ha^−1^ from 10 to 30 DAA, whereas reduced *C*_i_ was also observed. It was reasonable to conclude that the enhanced *A*_max_ drove the improved RUE and biomass production after anthesis. The greater leaf *G*_s_ under DF allowed more CO_2_ to be transported into the leaf. Even so, the *C*_i_ of flag leaves under DF was lower under DF. These results indicated that DF not only strengthened CO_2_ assimilating capacity but also expanded the ability to obtain CO_2_, which was in line with Wang et al. [57]. Importantly, the values of *A*_max_, *G*_s_, and *C*_i_ at T80 under DF were equivalent to those at T160 under FB. This indicated that DF combined with moderate NTR could achieve high *A*_max_ and produce more photosynthetic products. Sinclair and Horie [58] reported that RUE was closely linked to the leaf carbon exchange rate (i.e., *A*_max_), and the sensitivity of *A*_max_ to SLN is considered a potentially important source of variations in RUE. In the present study, from 10 to 30 DAA, DF consistently and significantly increased SLN at NTRs of 40–160 kg N ha^−1^. In addition, DF increased the CAT and SOD activities, which optimized leaf oxidation processes and protected the cells from oxidative damage, as well as reductions in the MDA content, thus improving the antioxidant capacity (Figures S2 and S3). These results indicate that DF produced a higher photosynthetic capacity in flag leaves by maintaining a better leaf N status and delaying leaf senescence after anthesis, thereby increasing the post-anthesis RUE of winter wheat.

Many studies have shown that the grain protein composition and contents and processing quality are strongly influenced by irrigation and fertilization practices [59,60,61]. The average grain protein content was significantly higher under DF than FB due to the improved availability of exogenous N and the reduced irrigation amount (decreasing from 60 to 40 mm per irrigation dose), as recognized by Coventry et al. [62] and Tari [63]. Importantly, increasing NTR was beneficial for enhancing the protein content and processing quality, as also shown by Zhao et al. [38], Ma et al. [40], and Guo et al. [41], but their responses to NTR differed between DF and FB. As NTR increased from 80 to 160 kg ha^−1^, the improvements in the total protein contents under DF (15.2–15.3%) were clearly greater than under FB (7.2–8.0%). The increases in the total protein content were attributed to increases in the gliadin and glutenin contents rather than the albumin and globulin contents, possibly because the accumulation of albumin and globulin depended mainly on the genotype, and the exogenous N supply had little effect [64,65]. The synthesis and accumulation of gliadin and glutenin mostly occur in the middle and late grain filling stages, and they are associated with the flag leaf N status and synthesis of free amino acids, which are determined by the available soil N supply [66]. In the present study, the SLN of flag leaves after anthesis was higher at T120 and T160 than T80 under DF. However, the higher SLN did not enhance *A*_max_, RUE, and the biomass production after anthesis, and further increased the yield compared with T80 under DF. It is reasonable to consider that the SLN reached a critical value at T80 and that the extra gains in SLN were mainly attributable to leaf N reserves and enhanced grain protein synthesis rather than the promotion of the photosynthetic capacity [52,67,68]. Gliadin and glutelin, constituting the main body of gluten, influence the processing quality of wheat grains [69,70]. Therefore, the responses of the processing quality traits to NRT were like those of the gliadin and glutenin contents in each season. In particular, the wet gluten content, gluten index, dough development, and stability time were 9.4–31.6% higher at T160 than T80 under DF, but only 2.3–7.1% higher under FB. Thus, DF enhanced the responses of the yield and quality to NTR compared with FB. The high yield was achieved at N topdressing at only 80 kg ha^−1^ under DF, but further increasing the N topdressing input improved the grain protein content and processing quality.

## 4. Materials and Methods

### 4.1. Site Description

During the 2021–2022 and 2022–2023 winter wheat growing seasons, a field experiment was conducted at Gucheng Village (35°43′ N, 111°44′ E), Yicheng County, Shanxi Province, China. The study site has a typical semi-arid warm temperate continental monsoon climate, and winter wheat and summer maize rotation is the main planting system. Experiments in both growing seasons were conducted in the same field and the former crop was summer maize with normal and uniform management. Five replicate topsoil (0–30 cm layer) samples were randomly collected for the determination of the soil’s basic nutrients before sowing in 2021 and 2022. The soil type was silty clay loam in the experimental site, and the organic matter, total N, alkaline N, Olsen P, available K, and pH were 19.11–20.07 g kg^−1^, 0.91–1.01 g kg^−1^, 47.25–51.21 mg kg^−1^, 19.71–20.44 mg kg^−1^, 168.22–171.17 mg kg^−1^, and 8.43–8.49, respectively.

**Figure 10:**
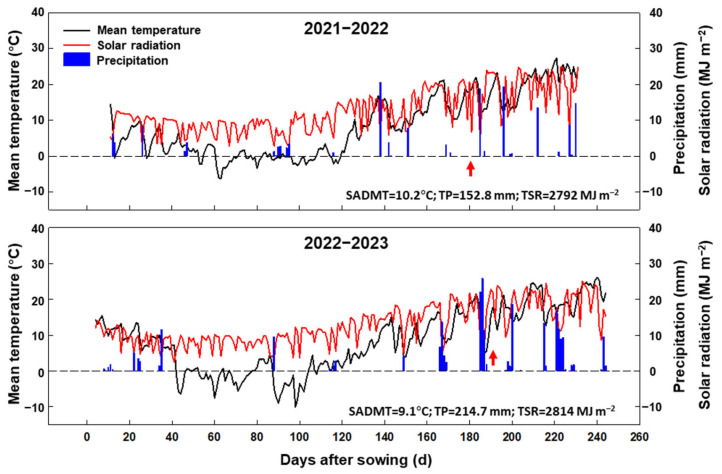
Daily mean temperatures, solar radiation, and precipitation recorded from sowing to maturity in the 2021–2022 and 2022–2023 growing seasons. The upward arrow indicates the date of anthesis. SADMT, seasonal average daily mean temperature; TP, total precipitation; TSR, total solar radiation.

In both growing seasons, climate parameters including the daily maximum temperature, minimum temperature, precipitation, and incident solar radiation were collected during the growing period from sowing to maturity by a weather station (AWS 800, Campbell Scientific, Inc., Logan, UT, USA) located about 50 m from the experimental field. Averaged over the last two decades, the seasonal average daily mean temperature, total precipitation, and total incident solar radiation during the winter wheat growing seasons (from 15 October to 20 June) were 9.5 °C, 182.4 mm, and 2803 MJ m^−2^, respectively. The seasonal average daily mean temperature, total precipitation, and incident solar radiation were 10.2 and 9.1 °C, 152.8 and 214.7 mm, and 2792 and 2814 MJ m^−2^ in the growing seasons in 2021–2022 and 2022–2023, respectively (Figure 10). The two experimental seasons did not display obvious differences compared to the seasons of the last two decades.

### 4.2. Experimental Design and Field Management

A newly released and high-yielding variety (Yannong1212) of winter wheat (*Triticum aestivum* L.) was sown during the two consecutive wheat growing seasons. Seeds were planted by using a wide-range sowing machine (2BMYF-10/5; Yuncheng Gongli Co., Ltd., Heze, China). The sowing belt was 10 cm with row spacing of 25 cm. The sowing machine has been widely used in Shanxi province. Wheat crops were sown on 4 November 2021 and 22 October 2022. The plant densities were 400 and 300 plants m^−2^ in 2021–2022 and 2022–2023, respectively. In 2021, heavy rain during early October led to late winter wheat sowing, and thus the higher seed sowing rate in the first season aimed to compensate for the yield loss due to delayed sowing.

The treatments were laid out in a split-plot design. The main plot used management practices, where the practices comprised FB and DF. Both practices used flood irrigation with 60 mm of water before the wintering stage. In the 2021–2022 growing season, FB was irrigated with 60 mm in the jointing stage, booting stage, and filling stage, whereas DF was irrigated with 40 mm. In general, winter wheat has a water requirement of 400–500 mm throughout its entire growth period, but our experimental site has an average annual precipitation of only 182.4 mm. Local farmers usually irrigated 3–4 times to meet the water requirement, each time with about 60 mm. DF is a water-saving and efficient technology, so our experiment set the DF treatment to reduce the irrigation amount by 1/3 on the basis of farmers. In the 2022–2023 growing season, due to the high precipitation in May, no irrigation was applied in the filling stage under either practice. The sub-plot was N treatment, where the six N treatments comprised N0 (no N applied), T0 (no N topdressing), T40 (N topdressing at 40 kg ha^−1^), T80 (N topdressing at 80 kg ha^−1^), T120 (N topdressing at 120 kg ha^−1^), and T160 (N topdressing at 160 kg ha^−1^). Except for N0, the other N topdressing treatments received 150 kg N ha^−1^ (urea, 46% N), 150 kg P_2_O_5_ ha^−1^ (calcium superphosphate, 16% P_2_O_5_), and 90 kg K_2_O ha^−1^ (potassium chloride, 52% K_2_O) before sowing, and each N topdressing treatment (urea, 46% N) was conducted at the jointing stage. Each plot had a length of 10.0 m and width of 2.0 m. Twelve (2 × 6) treatments were tested and each experimental treatment was replicated three times. The specific water and N management practices during the experimental periods are shown in Table S1.

A drip irrigation system was installed after wheat emergence. The drip tapes (Φ16 mm) were arranged 50 cm apart, with one drip line serving two rows of winter wheat, and the dripper spacing was 30 cm. The drippers discharged 2.2 L h^−1^ at a working pressure of 0.10–0.15 MPa. A flow meter was placed in each plot to monitor the amount of irrigation water released. At the jointing stage, the weighed urea was dissolved into the fertilizer tank, a Venturi fertilizer applicator was used to suck the fertilizer into the pipeline, and then it was dripped into the field with drip tapes. The same amount of water was added to the fertilization tanks in each plot.

### 4.3. Sampling and Measurements

#### 4.3.1. Grain Yield and Yield Components

At maturity, wheat spikes were cut from an area of 3.0 m^2^ (four typical rows with a length of 3.00 m) in the center of each plot to record the number of effective spikes that produced more than 5 grains per spike. After threshing the harvested spikes, the grains’ moisture content was measured using a digital moisture tester (PM8188A, Kett Electric Laboratory, Tokyo, Japan). The grain yield was corrected to 13.0% standard moisture content. Three 50.00 g sub-samples were weighed from the grain samples harvested in each plot to count the number of grains and to calculate the grain weight, which was also corrected to a 13.0% moisture content. The number of grains per spike was calculated as follows.

$$\mathrm{Grains}\;\mathrm{per}\;\mathrm{spike}=\frac{\mathrm{Grain}\;\mathrm{yield}}{\mathrm{Grain}\;\mathrm{weight}\times \mathrm{spike}\;\mathrm{number}}$$

#### 4.3.2. Biomass

At anthesis and maturity, the plants with a length of 0.5 m (0.125 m^2^) were randomly selected in the central row of each plot for sampling. The fresh samples initially kept for 0.5 h in an oven, and were then dried at 80 °C until they reached a constant weight. The plant samples taken at maturity were divided into grains and straws. The dry weight at anthesis was the pre-anthesis biomass production (BP_pre_), and the total biomass at maturity was the total dry weight of grain and straw. The post-anthesis biomass production (BP_post_) and biomass translocation (B_trans_) were calculated as follows.

$${\mathrm{BP}}_{\mathrm{post}}\;\left({\mathrm{kg}\;\mathrm{ha}}^{-1}\right)=\mathrm{Total}\;\mathrm{biomass}-{\mathrm{BP}}_{\mathrm{pre}}$$

$$B_{\mathrm{trans}}\;\left({\mathrm{kg}\;\mathrm{ha}}^{-1}\right)=\mathrm{Grain}\;\mathrm{biomass}-{\mathrm{BP}}_{\mathrm{post}}$$

The concentration of non-structural carbohydrates (NSC; soluble sugars and starch) was determined in straw at anthesis according to Yoshida [71]. Aliquots of the soluble sugar and starch extract were assayed by anthrone reagent colorimetry using a spectrophotometer (L8, Shanghai Yidian Analytical Instrument Co., Ltd., Shanghai, China) at a wavelength of 620 nm. The starch content was calculated by multiplying the glucose content by a conversion factor of 0.9 [72].

$$\mathrm{NSC}\;\mathrm{concentration}\;\left(\mathrm{mg}/g\right)=\mathrm{soluble}\;\mathrm{sugars}\;\mathrm{content}+\mathrm{starch}\;\mathrm{content}$$

$$\mathrm{NSC}\;\mathrm{accumulation}\;\mathrm{at}\;\mathrm{anthesis}\;\left({t\;\mathrm{ha}}^{-1}\right)=\mathrm{NSC}\;\mathrm{concentration}\times {\mathrm{BP}}_{\mathrm{pre}}$$

$$\mathrm{NSC}\;\mathrm{output}\;\mathrm{rate}\;\left(\%\right)=\frac{B_{\mathrm{trans}}}{\mathrm{NSC}\;\mathrm{concentration}}\times 100$$

The harvest index (HI) was calculated as follows.

$$\mathrm{HI}\;\left(\%\right)=\frac{\mathrm{Yield}\times 0.87}{\mathrm{Total}\;\mathrm{biomass}}\times 100$$

#### 4.3.3. Maximum Number of Stems and LAI

At the jointing stage, 1 m^2^ (a length of 1 m) of plants are selected from the center of each plot to count the total number of main stems and tillers (i.e., the maximum number of stems). The ratio of the spike number at maturity relative to the maximum number of stems at jointing is the percentage of effective stems. At anthesis, the wheat plants were sampled in the center of the plot with a length of 0.5 m (0.125 m^2^). All green leaves were separated and tiled on transparent polyethylene film to measure LAI using a leaf area meter (LI-3100C, LI-COR, Lincoln, NE, USA). LAI was calculated as follows.

$$\mathrm{LAI}\;\left(m^{2}{\;m}^{-2}\right)=\frac{\mathrm{Sampled}\;\mathrm{leaf}\;\mathrm{aera}}{\mathrm{Sampled}\;\mathrm{land}\;\mathrm{area}}$$

#### 4.3.4. SLN

After measuring the LAI, the leaves were dried in an oven at 80 °C to constant weight and then crushed for measurement. The leaf N concentration (N mass per unit dry weight, mg g^−1^) was determined using an elemental analyzer (Rapid N Exceed, Elementar, Langenselbold, Germany). SLN (g m^−2^) was calculated as follows.

$$\mathrm{SLN}\;\left({g\;m}^{-2}\right)=\frac{\mathrm{Leaf}\;N\;\mathrm{concentration}\times \mathrm{Leaf}\;\mathrm{mass}}{\mathrm{Sampled}\;\mathrm{leaf}\;\mathrm{aera}}$$

#### 4.3.5. Light Interception and Radiation Use Efficiency

The canopy PAR interception ratio was measured at an interval of 10–15 days during the growing season. The measurements were conducted between 1100 and 1300 h using a linear PAR ceptometer (AccuPAR LP-80, Decagon Devices Inc., Pullman, WA, USA). In each plot, the light bar was placed vertically to the rows and slightly above the soil surface, and then the transmitted PAR intensity was measured. After the measurement of the transmitted PAR intensity, the PAR intensity was immediately recorded above the canopy. Six pairs of PAR intensity measurements were recorded. The canopy PAR interception ratio (PARI) was calculated as follows.

$$\mathrm{PARI}\;\left(\%\right)=\frac{\mathrm{PAR}\;\mathrm{intensity}\;\mathrm{above}\;\mathrm{canopy}-\mathrm{PAR}\;\mathrm{intensity}\;\mathrm{slightly}\;\mathrm{above}\;\mathrm{the}\;\mathrm{soil}\;\mathrm{surface}\;}{\mathrm{PAR}\;\mathrm{intensity}\;\mathrm{above}\;\mathrm{canopy}}\times 100$$

The intercepted solar radiation (ISR) during the growth period was calculated using the average canopy PARI and accumulated incident solar radiation during the growth period, as follows.

$$\mathrm{ISR}\;\left({\mathrm{MJ}\;m}^{-2}\right)=\frac{\mathrm{PARI}\;\mathrm{at}\;\mathrm{the}\;\mathrm{beginning}+\mathrm{PARI}\;\mathrm{at}\;\mathrm{the}\;\mathrm{end}\;\mathrm{of}\;\mathrm{the}\;\mathrm{period}}{2}\times \mathrm{incident}\;\mathrm{solar}\;\mathrm{radiation}$$

The ISR during the entire growing season was the sum of the ISR values during each growth period. The RUE during one period was calculated as follows.

$$\mathrm{RUE}\;\left({g\;\mathrm{MJ}}^{-1}\right)=\frac{\mathrm{Biomass}}{\mathrm{ISR}}$$

#### 4.3.6. Photosynthetic Characteristics

Light-saturated photosynthetic rate (*A*_max_), stomatal conductance (*G*_s_), and intercellular CO_2_ concentration (*C*_i_) were measured for flag leaves using a Li-Cor LI-6400XT Portable Photosynthesis System (Licor Biosciences, Lincoln, NE, USA) at anthesis, 10 days after anthesis (10 DAA), 20 DAA, and 30 DAA. The instrument was adjusted for humidity at 50–60% within the cuvette and the settings used for obtaining the *A*_max_ readings were PAR intensity = 1500 μmol m^−2^ s^−1^, CO_2_ concentration in sample chamber = 360 μL L^−1^, and gas flow rate = 400 μmol s^−1^. The measurements were taken between 10:00 and 15:00 [73].

#### 4.3.7. Catalase (CAT) and Superoxide Dismutase (SOD) Activities, and Malondialdehyde (MDA) Contents

Twenty flag leaves were sampled from each plot at anthesis, 10 DAA, 20 DAA, and 30 DAA. Fresh samples were immediately immersed in liquid N and then stored in an ultra-low temperature refrigerator at −80 °C until biochemical determination. Each fresh flag leaf was used to determine the antioxidant enzyme activities and MDA content. We used the methods described by Wang and Huang [74] to measure the CAT activity, SOD activity, and MDA content. The CAT activity was determined based on the consumption of H_2_O_2_ at 240 nm during 3 min. The SOD activity was evaluated based on its ability to inhibit the photoreduction of nitroblue tetrazolium. The MDA content was determined based on the thiobarbituric acid reaction [75].

#### 4.3.8. Protein Content of Grains and Protein Components

Osborne protein fractions were extracted from wholemeal flour using a sequential extraction procedure according to a previous study [76]. First, one gram of wholemeal flour was weighed to extract the structural or metabolic proteins (albumin and globulin) with a mixed solution (0.067 mol L^−1^ HKNaPO_4_, pH 7.6, containing 0.4 mol L^−1^ NaCl, 0.067 mol L^−1^ Na_2_HPO_4_/KH_2_PO_4_) in a plastic centrifuge tube. The supernatant was collected as the albumin and globulin fraction after two rounds of oscillation and centrifugation. The residue in the tube was extracted three times with 70% ethanol to obtain the wholemeal flour gliadin. The upper residue was extracted with another mixed solution to obtain the final fraction of glutenin using the same procedure described above, which was repeated twice. The concentration of the wholemeal flour protein fraction was determined by using the Biuret method at 480 nm (UV-2450, Shimadzu, Kyoto, Japan).

#### 4.3.9. Grain Quality

The wet gluten content and gluten index were determined by using a 2200 gluten washing instrument (Perten, Alpnach, Switzerland) according to standards GB/T 14608-1993 [77] and LS/T 6102-1995 [78]. The farinograph parameters (dough development time and stability time) were determined by using a FarinoGraph-E (Brabender, Duisburg, Germany) according to standard GB/T 14614-2019 [79]. 

### 4.4. Data Analysis

Statistical data analyses were performed with Statistix 9.0 (Analytical Software, Tallahassee, FL, USA). Two-way analysis of variance (ANOVA) was conducted using a mixed general linear model in each year. Treatment means were compared with the least significant difference test (*α* = 0.05). Differences in yields and yield-related traits under DF and FB at the same NTR were assessed using Student’s *t*-test (*α* = 0.05). Pearson’s correlation coefficients were used to analyze the relationships between traits. Plotting was performed using SigmaPlot 12.5 (Systat Software Inc., Point Richmond, CA, USA) software.

## 5. Conclusions

This field experiment conducted over two seasons confirmed our hypothesis that DF would enhance the responses of the yield and quality to NRT. The higher flag leaf photosynthetic capacity after anthesis due to the enhanced SLN and antioxidant capacity under DF improved the post-anthesis RUE and biomass production, thereby improving the wheat yield. The medium NTR of 80 kg ha^−1^ produced the highest yield under DF, but further increasing NTR enhanced the protein content, gluten characteristics, and processing quality. These findings may help to optimize N fertilizer management strategies for winter wheat production with drip fertigation in north China.

## Figures and Tables

**Table 1:** Yield components, maximum stem number, productive stem percentage, and leaf area index (LAI) at the anthesis of winter wheat in the 2021–2022 and 2022–2023 growing seasons.

Growing Season	Management Pattern	N Treatment	Spikes Number	Grains per Spike	Grain Weight	Maximum Stem Number	Productive Stem Percentage	LAI at Anthesis
			(m^–2^)		(mg)	(m^–2^)	(%)	(m^2^ m^–2^)
2021–2022	FB	N0	435.0 e	29.9 e	53.9 a	1356 b	32.1 a	4.21 d
		T0	517.6 d	34.4 d	51.3 b	1985 a	26.1 d	5.55 c
		T40	555.8 c	36.6 c	49.9 c	1973 a	28.2 c	6.06 b
		T80	590.2 b	37.6 b	49.3 cd	1986 a	29.7 b	6.47 a
		T120	611.6 ab	38.5 ab	48.6 d	1927 a	31.7 a	6.56 a
		T160	620.0 a	39.2 a	48.2 d	1989 a	31.2 ab	6.62 a
		**Mean**	**555.0 B**	**36.0 A**	**50.2 B**	**1869 A**	**29.8 B**	**5.91 A**
	DF	N0	439.0 d	29.0 d	54.6 a	1319 b	33.3 a	4.15 d
		T0	531.7 c	33.4 c	52.4 ab	1965 a	27.1 c	5.70 c
		T40	589.3 b	35.0 b	52.8 b	1912 a	30.8 b	6.26 b
		T80	634.3 a	36.4 a	52.4 b	1927 a	32.9 a	6.78 a
		T120	649.3 a	36.5 a	51.5 c	1909 a	34.0 a	6.84 a
		T160	653.0 a	37.2 a	50.2 d	1974 a	33.1 a	6.83 a
		**Mean**	**582.8 A**	**34.6 A**	**52.3 A**	**1834 A**	**31.5 A**	**6.09 A**
	ANOVA							
	P		**	ns	**	ns	**	ns
	N		**	**	**	**	**	**
	P × N		**	*	**	ns	*	ns
2022–2023	FB	N0	460.0 e	28.7 e	50.9 a	1499 b	30.7 a	4.30 e
		T0	552.5 d	32.2 d	48.1 b	2190 a	25.2 d	5.73 d
		T40	586.1 c	34.0 c	46.9 c	2213 a	26.5 c	6.16 c
		T80	611.8 b	36.6 b	45.5 d	2197 a	27.9 b	6.62 a
		T120	637.8 ab	38.0 a	45.0 d	2196 a	29.0 ab	6.74 a
		T160	657.4 a	38.5 a	44.7 d	2181 a	30.1 a	6.90 a
		**Mean**	**583.3 B**	**34.7 A**	**46.9 B**	**2079 A**	**28.2 B**	**6.07 A**
	DF	N0	457.7 d	28.8 d	51.1 a	1506 b	30.4 b	4.27 d
		T0	572.1 c	30.8 c	49.4 b	2210 a	25.9 d	5.89 c
		T40	628.6 b	33.2 b	48.9 b	2162 a	29.1 c	6.41 b
		T80	671.0 a	35.2 a	48.5 b	2160 a	31.1 ab	6.85 a
		T120	676.5 a	36.0 a	47.8 c	2151 a	31.4 ab	6.95 a
		T160	688.6 a	36.1 a	46.7 d	2132 a	32.3 a	7.03 a
		**Mean**	**615.7 A**	**33.4 A**	**48.7 A**	**2053 A**	**30.0 A**	**6.23 A**
	ANOVA							
	P		**	ns	**	ns	**	ns
	N		**	**	**	**	**	**
	P × N		**	*	**	ns	*	ns

Within a column for each growing season, means followed by different uppercase letters are significantly different according to the least significant difference test (LSD, α = 0.05) between two management patterns. Within a column for management pattern, means followed by different lowercase letters are significantly different according to the least significant difference test (LSD, α = 0.05) among six N treatments in each season. Here, * and ** indicate significance at *p* = 0.05 and 0.01, respectively; ns is insignificant at *p* = 0.05. FB and DF refer to flood irrigation and broadcast fertilizer and drip fertigation, respectively. N0, T0, T40, T80, T120, and T160 indicate the N omission and N topdressing rates of 0, 40, 80, 120 and 160 kg ha^−1^, respectively.

**Table 2:** Translocation and accumulation of pre-anthesis non-structural carbohydrate (NSC), biomass, and harvest index of winter wheat in the 2021–2022 and 2022–2023 growing seasons.

Growing Season	Management Pattern	N Treatment	NSC Accumulation at Anthesis	Biomass Translocation	NSC Output Rate	The Contribution Rate of Biomass Translocation to Grain	Harvest Index
			(t ha^–1^)	(t ha^–1^)	(%)	(%)	(%)
2021–2022	FB	N0	2.13 e	1.47 b	69.2 a	24.2 a	46.9 a
		T0	3.09 d	2.02 a	65.3 b	25.5 a	46.8 a
		T40	3.59 c	2.19 a	60.9 c	24.7 a	46.6 a
		T80	4.07 b	2.05 a	50.2 d	21.5 b	46.1 ab
		T120	4.46 a	1.99 a	44.8 e	20.0 b	45.8 ab
		T160	4.66 a	2.06 a	44.1 e	20.2 b	45.3 b
		**Mean**	**3.67 A**	**1.96 A**	**55.8 A**	**22.7 A**	**46.2 A**
	DF	N0	1.97 e	1.33 c	67.5 a	22.1 a	46.1 a
		T0	3.14 d	1.92 a	60.6 b	23.4 a	46.5 a
		T40	3.82 c	2.09 a	54.6 c	22.0 a	46.5 a
		T80	4.43 b	1.79 ab	40.3 d	17.0 b	45.9 a
		T120	4.63 ab	1.83 ab	39.5 d	17.2 b	45.9 a
		T160	4.77 a	1.68 b	35.2 e	15.8 b	45.3 a
		**Mean**	**3.79 A**	**1.77 B**	**49.6 B**	**19.6 B**	**46.0 A**
	ANOVA						
	P		ns	*	**	**	ns
	N		**	**	**	**	*
	P × N		**	**	**	**	ns
2022–2023	FB	N0	2.67 f	1.60 b	59.8 a	27.3 a	48.1 a
		T0	3.42 e	2.00 a	58.4 a	26.8 a	46.5 b
		T40	4.01 d	2.03 a	50.6 b	24.9 b	45.4 bc
		T80	4.51 c	1.96 a	43.4 c	22.1 c	44.4 c
		T120	4.88 b	2.11 a	43.2 c	22.2 c	45.0 bc
		T160	5.17 a	2.17 a	41.9 c	22.0 c	44.8 bc
		**Mean**	**4.11 A**	**1.98 A**	**49.5 A**	**24.2 A**	**45.7 A**
	DF	N0	2.69 e	1.52 b	54.2 a	25.7 a	47.7 a
		T0	3.53 d	1.95 a	51.6 b	25.3 a	46.3 b
		T40	4.36 c	2.08 a	47.7 c	23.4 b	45.4 bc
		T80	4.96 b	1.99 a	40.1 d	20.0 c	45.1 c
		T120	5.11 ab	1.96 a	38.3 de	19.3 c	44.8 c
		T160	5.30 a	1.86 a	35.0 e	18.3 d	44.5 c
		**Mean**	**4.33 A**	**1.89 A**	**44.5 B**	**22.0 B**	**45.6 A**
	ANOVA						
	P		ns	ns	**	**	ns
	N		**	**	**	**	**
	P × N		**	**	**	**	ns

Within a column for each growing season, means followed by different uppercase letters are significantly different according to the least significant difference test (LSD, *α* = 0.05) between two management patterns. Within a column for management pattern, means followed by different lowercase letters are significantly different according to the least significant difference test (LSD, α = 0.05) among six N treatments in each season. Here, * and ** indicate significance at *p* = 0.05 and 0.01, respectively; ns is insignificant at *p* = 0.05. FB and DF refer to flood irrigation and broadcast fertilizer and drip fertigation, respectively. N0, T0, T40, T80, T120, and T160 indicate the N omission and N topdressing rates of 0, 40, 80, 120, and 160 kg ha^−1^, respectively.

**Table 3:** Grain protein contents and protein components of winter wheat in the 2021–2022 and 2022–2023 growing seasons.

Growing Season	Management Pattern	N Treatment	Protein Components (%)			Total Protein (%)
			Albumin + Globulin	Gliadin	Glutenin	
2021–2022	FB	N0	3.18 a	3.62 c	4.86 c	11.66 c
		T0	3.22 a	3.81 bc	5.04 bc	12.07 bc
		T40	3.24 a	4.03 ab	5.26 ab	12.53 ab
		T80	3.26 a	4.16 a	5.39 a	12.81 a
		T120	3.28 a	4.20 a	5.43 a	12.91 a
		T160	3.27 a	4.21 a	5.46 a	12.94 a
		**Mean**	**3.25 A**	**4.08 B**	**5.32 B**	**12.65 B**
	DF	N0	3.22 a	3.70 e	4.95 d	11.87 e
		T0	3.26 a	3.87 de	5.05 d	12.18 de
		T40	3.25 a	4.09 cd	5.39 c	12.73 cd
		T80	3.27 a	4.31 bc	5.59 bc	13.17 bc
		T120	3.30 a	4.51 ab	5.80 ab	13.61 ab
		T160	3.28 a	4.72 a	6.03 a	14.03 a
		**Mean**	**3.27 A**	**4.30 A**	**5.57 A**	**13.15 A**
	ANOVA					
	P		ns	**	**	**
	N		ns	**	**	**
	P × N		ns	**	**	**
2022–2023	FB	N0	2.95 a	3.52 c	4.57 c	11.04 c
		T0	3.00 a	3.71 bc	4.78 bc	11.49 bc
		T40	3.03 a	3.88 ab	5.00 ab	11.91 ab
		T80	3.05 a	4.03 a	5.15 a	12.23 a
		T120	3.07 a	4.07 a	5.20 a	12.34 a
		T160	3.06 a	4.11 a	5.24 a	12.41 a
		**Mean**	**3.04 A**	**3.96 B**	**5.07 B**	**12.08 B**
	DF	N0	2.98 a	3.60 e	4.66 e	11.24 e
		T0	3.00 a	3.80 de	4.90 de	11.71 de
		T40	3.01 a	4.01 cd	5.08 cd	12.09 cd
		T80	3.05 a	4.18 bc	5.31 bc	12.54 bc
		T120	3.07 a	4.39 b	5.57 ab	13.03 ab
		T160	3.11 a	4.63 a	5.76 a	13.50 a
		**Mean**	**3.05 A**	**4.20 A**	**5.33 A**	**12.58 A**
	ANOVA					
	P		ns	**	**	**
	N		ns	**	**	**
	P × N		ns	**	**	**

Within a column for each growing season, means followed by different uppercase letters are significantly different according to the least significant difference test (LSD, *α* = 0.05) between two management patterns. Within a column for management pattern, means followed by different lowercase letters are significantly different according to the least significant difference test (LSD, α = 0.05) among six N treatments in each season. Here, ** is significant at *p* = 0.05; ns is insignificant at *p* = 0.05. FB and DF refer to flood irrigation and broadcast fertilizer and drip fertigation, respectively. N0, T0, T40, T80, T120, and T160 indicate the N omission and N topdressing rates of 0, 40, 80, 120, and 160 kg ha^−1^, respectively.

## Data Availability

Data are contained within the article.

## Supplementary Materials

The following supporting information can be downloaded at: https://www.mdpi.com/article/10.3390/plants13111439/s1, Table S1: Irrigation amount and nitrogen fertilization rate at different growing stages of winter wheat in the 2021–2022 and 2022–2023 growing seasons; Figure S1: The CAT and SOD activities and MDA contents of flag leaves after anthesis in the 2021–2022 growing season; Figure S2: The CAT and SOD activities and MDA contents of flag leaves after anthesis in the 2022–2023 growing season.

## References

[1] FAO (Food and Agriculture Organization) (2023). Online Statistical Database: Crops and Livestock Products. https://www.fao.org/faostat/en/#data/QCL.

[2] National Bureau of Statistics of China (2022). China Statistical Yearbook.

[3] Kang S.Z., Zhang L., Liang Y.L., Hu X.T., Cai H.J., Gu B.T. (2002). Effects of limited irrigation on yield and water use efficiency of winter wheat in the Loess Plateau of China. Agric. Water Manag..

[4] Dang J.Y., Pei X.X., Zhang D.Y., Zhang J., Cheng M.F., Wang J.A., Gao L. (2020). Effects of integration of micro-sprinkler irrigation and nitrogen on growth and development of winter wheat and water and fertilizer use efficiency. Chin. J. Appl. Ecol..

[5] Peng S.B., Bouman B., Visperas R.M., Castañeda A., Nie L.X., Park H.K. (2006). Comparison between aerobic and flooded rice in the tropics: Agronomic performance in an eight-season experiment. Field Crops Res..

[6] Dalin C., Wada Y., Kastner T., Puma M.J. (2017). Groundwater depletion embedded in international food trade. Nature.

[7] Shahdany S.M.H., Firoozfar A., Maestre J.M., Mallakpour I., Taghvaeian S., Karimi P. (2018). Operational performance improvements in irrigation canals to overcome groundwater overexploitation. Agric. Water Manag..

[8] Uhart S.A., Andrade F.H. (1995). Nitrogen deficiency in maize I. Effects on crop growth, development, dry matter partitioning, and kernel set. Crop Sci..

[9] Prystupa P., Slafer G.A., Savin R. (2003). Leaf appearance, tillering and their coordination in response to NxP fertilization in barley. Plant Soil.

[10] Yang D.Q., Cai T., Luo Y.L., Wang Z.L. (2019). Optimizing plant density and nitrogen application to manipulate tiller growth and increase grain yield and nitrogen-use efficiency in winter wheat. PeerJ.

[11] Matson P.A., Naylor R., Ortiz-Monasterio I. (1998). Integration of environmental, agronomic, and economic aspects of fertilizer management. Science.

[12] Dar E.A., Brar A.S., Mishra S.K., Singh K.B. (2017). Simulating response of wheat to timing and depth of irrigation water in drip irrigation system using CERES-wheat model. Field Crops Res..

[13] Lv H.F., Zhao Y.M., Wang Y.F., Wan L., Wang J.G., Butterbach-Bahl K., Lin S. (2020). Conventional flooding irrigation and over fertilization drives soil pH decrease not only in the top-but also in subsoil layers in solar greenhouse vegetable production systems. Geoderma.

[14] Lu J.S., Xiang Y.Z., Fan J.L., Zhang F.C., Hu T.T. (2021). Sustainable high grain yield, nitrogen use efficiency and water productivity can be achieved in wheat-maize rotation system by changing irrigation and fertilization strategy. Agric. Water Manag..

[15] Si Z.Y., Zain M., Mehmood F., Wang G.S., Gao Y., Duan A.W. (2020). Effects of nitrogen application rate and irrigation regime on growth, yield, and water-nitrogen use efficiency of drip-irrigated winter wheat in the North China Plain. Agric. Water Manag..

[16] Yan F.L., Zhang F.C., Fan X.K., Fan J.L., Wang Y., Zou H.Y., Wang H.D., Li G.D. (2021). Determining irrigation amount and fertilization rate to simultaneously optimize grain yield, grain nitrogen accumulation and economic benefit of drip-fertigated spring maize in northwest China. Agric. Water Manag..

[17] Hagin J., Lowengart A. (1995). Fertigation for minimizing environmental pollution by fertilizers. Fertil. Res..

[18] Fan J.L., Wu L.F., Zhang F.C., Yan S.C., Xiang Y.Z. (2017). Evaluation of drip fertigation uniformity affected by injector type, pressure difference and lateral layout. Irrig. Drain..

[19] Yan S.C., Wu Y., Fan J.L., Zhang F.C., Guo J.J., Zheng J., Wu L.F. (2022). Quantifying grain yield, protein, nutrient uptake and utilization of winter wheat under various drip fertigation regimes. Agric. Water Manag..

[20] Wang X.K., Yun J., Shi P., Li Z.B., Li P., Xing Y.Y. (2019). Root growth, fruit yield and water use efficiency of greenhouse grown tomato under different irrigation regimes and nitrogen levels. J. Plant Growth Regul..

[21] Fanish S.A., Muthukrishnan P., Santhi P. (2011). Effect of drip fertigation on field crops—A review. Agric. Rev..

[22] Jha S.K., Ramatshaba T.S., Wang G.S., Liang Y., Liu H., Gao Y.P., Liu H., Gao Y., Duan A.W. (2019). Response of growth, yield and water use efficiency of winter wheat to different irrigation methods and scheduling in North China Plain. Agric. Water Manag..

[23] Soni J.K., Raja N.A., Kumar V. (2019). Improving productivity of groundnut (*Arachis hypogaea* L.) under drip and micro sprinkler fertigation system. Legume Res.-Int. J..

[24] Zheng J., Zhou M.H., Zhu B., Fan J.L., Lin H.Y., Ren B., Zhang F.C. (2023). Drip fertigation sustains crop productivity while mitigating reactive nitrogen losses in Chinese agricultural systems: Evidence from a meta-analysis. Sci. Total Environ..

[25] Delbaz R., Ebrahimian H., Abbasi F., Ghameshlou A.N., Liaghat A., Ranazadeh D. (2023). A global meta-analysis on surface and drip fertigation for annual crops under different fertilization levels. Agric. Water Manag..

[26] Li H.R., Mei X.R., Wang J.D., Huang F., Hao W.P., Li B.G. (2021). Drip fertigation significantly increased crop yield, water productivity and nitrogen use efficiency with respect to traditional irrigation and fertilization practices: A meta-analysis in China. Agric. Water Manag..

[27] Cassman K.G. (1999). Ecological intensification of cereal production systems: Yield potential, soil quality, and precision agriculture. Proc. Natl. Acad. Sci. USA.

[28] Parry M.A., Reynolds M., Salvucci M.E., Raines C., Andralojc P.J., Zhu X.G., Price G.D., Condon A.G., Furbank R.T. (2011). Raising yield potential of wheat. II. Increasing photosynthetic capacity and efficiency. J. Exp. Bot..

[29] Monteith J.L. (1972). Solar radiation and productivity in tropical ecosystems. J. Appl. Ecol..

[30] Monteith J.L. (1977). Climate and the efficiency of crop production in Britain. Philos. Trans. R. Soc. Lond. B Biol. Sci..

[31] Zhu X.G., Long S.P., Ort D.R. (2010). Improving photosynthetic efficiency for greater yield. Annu. Rev. Plant Boil..

[32] Allard V., Martre P., Le Gouis J. (2013). Genetic variability in biomass allocation to roots in wheat is mainly related to crop tillering dynamics and nitrogen status. Eur. J. Agron..

[33] Raymbek A., Saljnikov E., Kenenbayev S., Perovic V., Cakmak D., Ramazanova S. (2017). Protein content changes in wheat grain as influenced by nitrogen fertilization. Agrochimica.

[34] Sinclair T.R., Muchow R.C. (1999). Radiation use efficiency. Adv. Agron..

[35] Fletcher A.L., Johnstone P.R., Chakwizira E., Brown H.E. (2013). Radiation capture and radiation use efficiency in response to N supply for crop species with contrasting canopies. Field Crops Res..

[36] Ullah H., Santiago-Arenas R., Ferdous Z., Attia A., Datta A. (2019). Improving water use efficiency, nitrogen use efficiency, and radiation use efficiency in field crops under drought stress: A review. Adv. Agron..

[37] Wen M.X., Chen D.A., Li D.S., Qu Z.X., Cai J.H. (2014). Regulating effect of nitrogen topdressing and planting density on population quality, yield and quality of Zhenmai 168. J. Triticeae Crops.

[38] Zhao G.C., Chang X.H., Yang Y.S., Li Z.H., Feng M., Ma S.K., Yang G.X. (2010). Grain yield and quality responding to the nitrogen fertilizer operation in different quality type wheat. J. Plant Nutr. Fert..

[39] Ma R.Q., Tao Z.Q., Wang D.M., Wang Y.J., Yang Y.S., Nu Z.L., Zhao G.C., Chang X.H. (2020). Effects of topdressing nitrogen rate on photosynthetic characteristics and yield of flag leaves of wheat in different regions. J. Nucl. Agric. Sci..

[40] Ma R.Q., Tao Z.Q., Wang D.M., Wang Y.J., Yang Y.S., Zhu Y.J., Zhao K.N., Li J.Z., Wang Y.J., Chang X.H. (2019). Effect of nitrogen topdressing rate on yield and quality of medium and strong gluten wheat cultivars. J. Plant Nutr. Fert..

[41] Guo R., Huang X.G., Wen M.X., Chen C., Liu J.G., Shan Y.B., Qu Z.X., Li D.S. (2020). Effect of the nitrogen topdressing and plant density on grain yield and quality of spring strong gluten wheat cultivar zhenmai 12. J. Nucl. Agric. Sci..

[42] Zhang L., He X.R., Liang Z.Y., Zhang W., Zou C.Q., Chen X.P. (2020). Tiller development affected by nitrogen fertilization in a high-yielding wheat production system. Crop Sci..

[43] Yang H.K., Xiao Y., He P., Ai D.L., Zou Q.S., Hu J., Liu L., Huang X.L., Zheng T., Fan G.Q. (2022). Straw mulch-based no-tillage improves tillering capability of dryland wheat by reducing asymmetric competition between main stem and tillers. Crop J..

[44] Bai S.S., Kang Y.H., Wan S.Q. (2020). Drip fertigation regimes for winter wheat in the North China Plain. Agric. Water Manag..

[45] Hamani A.K.M., Abubakar S.A., Si Z.Y., Kama R., Gao Y., Duan A.W. (2023). Responses of grain yield and water-nitrogen dynamic of drip-irrigated winter wheat to different nitrogen fertigation and water regimes in the North China Plain. Agric. Water Manag..

[46] Dordas C. (2009). Dry matter, nitrogen and phosphorus accumulation, partitioning and remobilization as affected by N and P fertilization and source-sink relations. Eur. J. Agron..

[47] Manschadi A.M., Soltani A. (2021). Variation in traits contributing to improved use of nitrogen in wheat: Implications for genotype by environment interaction. Field Crops Res..

[48] Ercoli L., Lulli L., Mariotti M., Masoni A., Arduini I. (2008). Post-anthesis dry matter and nitrogen dynamics in durum wheat as affected by nitrogen supply and soil water availability. Eur. J. Agron..

[49] Duan J.Z., Wu Y.P., Zhou Y., Ren X.X., Shao Y.H., Feng W., Zhu Y.J., Wang Y.H., Guo T.C. (2018). Grain number responses to pre-anthesis dry matter and nitrogen in improving wheat yield in the Huang-Huai Plain. Sci. Rep..

[50] Liu M., Wu X.L., Li C.S., Li M., Xiong T., Tang Y.L. (2020). Dry matter and nitrogen accumulation, partitioning, and translocation in synthetic-derived wheat cultivars under nitrogen deficiency at the post-jointing stage. Field Crops Res..

[51] Li H.T., Shao L.W., Liu X.W., Sun H.Y., Chen S.Y., Zhang X.Y. (2023). What matters more, biomass accumulation or allocation, in yield and water productivity improvement for winter wheat during the past two decades?. Eur. J. Agron..

[52] Evans J.R. (1983). Nitrogen and photosynthesis in the flag leaf of wheat (*Triticum aestivum* L.). Plant Physiol..

[53] Carmo-Silva E., Andralojc P.J., Scales J.C., Driever S.M., Mead A., Lawson T., Raines C.A., Parry M.A. (2017). Phenotyping of field-grown wheat in the UK highlights contribution of light response of photosynthesis and flag leaf longevity to grain yield. J. Exp. Bot..

[54] Fan Y.H., Ma L.L., Yang J.H., Ding W.J., He W., Tang Y., Cui G.J., Zhang W.J., Ma S.Y., Ma C.X. (2023). Night warming from tillering to jointing increases post-anthesis flag leaf photosynthetic capacity and wheat yield. Eur. J. Agron..

[55] Zhang Y., Wang J., Gong S., Xu D., Mo Y., Zhang B. (2021). Straw mulching improves soil water content, increases flag leaf photosynthetic parameters and maintaines the yield of winter wheat with different irrigation amounts. Agric. Water Manag..

[56] Wang Y., Xu Y., Guo Q., Zhang P., Cai T., Jia Z. (2023). Adopting nitrogen deep placement based on different simulated precipitation year types enhances wheat yield and resource utilization by promoting photosynthesis capacity. Field Crops Res..

[57] Wang H.Q., Wang R.R., Jiang G.Y., Yin H.J., Yan S.J., Che Z.Q. (2023). Effect of amount of nitrogen fertilizer applied on photosynthetic physiological characteristics of drip irrigated spring wheat leaves. Acta Agron. Sin..

[58] Sinclair T.R., Horie T. (1989). Leaf nitrogen, photosynthesis, and crop radiation use efficiency: A review. Crop Sci..

[59] Wang C.Y., Liu W.X., Li Q.X., Ma D.Y., Lu H.F., Feng W., Xie Y.X., Zhu Y.J., Guo T.C. (2014). Effects of different irrigation and nitrogen regimes on root growth and its correlation with above-ground plant parts in high-yielding wheat under field conditions. Field Crops Res..

[60] Zhang P.P., Ma G., Wang C.Y., Lu H.F., Li S.S., Xie Y.X., Ma D.Y., Zhu Y.J., Guo T.C. (2017). Effect of irrigation and nitrogen application on grain amino acid composition and protein quality in winter wheat. PLoS ONE.

[61] Li Y., Huang G.H., Chen Z.J., Xiong Y.W., Huang Q.Z., Xu X., Huo Z.L. (2022). Effects of irrigation and fertilization on grain yield, water and nitrogen dynamics and their use efficiency of spring wheat farmland in an arid agricultural watershed of Northwest China. Agric. Water Manag..

[62] Coventry D.R., Yadav A., Poswal R.S., Sharma R.K., Gupta R.K., Chhokar R.S., Gill S.C., Kumar V., Kumar A., Mehta A. (2011). Irrigation and nitrogen scheduling as a requirement for optimising wheat yield and quality in Haryana, India. Field Crops Res..

[63] Tari A.F. (2016). The effects of different deficit irrigation strategies on yield, quality, and water-use efficiencies of wheat under semi-arid conditions. Agric. Water Manag..

[64] Wieser H., Seilmeier W. (1998). The influence of nitrogen fertilisation on quantities and proportions of different protein types in wheat flour. J. Sci. Food Agric..

[65] Reynolds M., Bonnett D., Chapman S.C., Furbank R.T., Manès Y., Mather D.E., Parry M.A. (2011). Raising yield potential of wheat. I. Overview of a consortium approach and breeding strategies. J. Exp. Bot..

[66] Cui H.X., Luo Y.L., Li C.H., Chang Y.L., Jin M., Li Y., Wang Z.L. (2023). Effects of nitrogen forms on nitrogen utilization, yield, and quality of two wheat varieties with different gluten characteristics. Eur. J. Agron..

[67] Millard P. (1988). The accumulation and storage of nitrogen by herbaceous plants. Plant Cell Environ..

[68] Pask A.J.D., Sylvester-Bradleyb R., Jamiesonc P.D., Foulkesa M.J. (2012). Quantifying how winter wheat crops accumulate and use nitrogen reserves during growth. Field Crops Res..

[69] Dier M., Hüther L., Schulze W.X., Erbs M., Köhler P., Weigel H.J., Manderscheid R., Zörb C. (2020). Elevated atmospheric CO_2_ concentration has limited effect on wheat grain quality regardless of nitrogen supply. J. Agric. Food Chem..

[70] Lyu X.K., Liu Y., Li N., Ku L.B., Hou Y.T., Wen X.X. (2022). Foliar applications of various nitrogen (N) forms to winter wheat affect grain protein accumulation and quality via N metabolism and remobilization. Crop J..

[71] Cock J.H., Yoshida S., Forno D.A. (1976). Laboratory Manual for Physiological Studies of Rice.

[72] Li X., Zhang R.C., Chen G., Xie J.X., Xiao Z.W., Cao F.B., Ali I., Anas I., Wahab A., Huang M. (2023). Increasing grain weight and yield stability by increasing pre-heading non-structural carbohydrate reserves per spikelet in short-growth duration rice. Crop J..

[73] Chen Z., Newman I., Zhou M., Mendham N., Zhang G., Shabala S. (2005). Screening plants for salt tolerance by measuring K+ flux: A case study for barley. Plant Cell Environ..

[74] Wang X.K., Huang J.L. (2000). Principles and Techniques of Plant Physiological Biochemical Experiment.

[75] Ahmad I., Kamran M., Yang X., Meng X., Ali S., Ahmad S., Zhang X., Bilegjargal B., Ahmad B., Liu T. (2019). Effects of applying uniconazole alone or combined with manganese on the photosynthetic efficiency, antioxidant defense system, and yield in wheat in semiarid regions. Agric. Water Manag..

[76] Luo L.C., Hui X.L., Wang Z.H., Zhang X., Xie Y.H., Gao Z.Q., Chai S.X., Lu Q.L., Li T.L., Sun M. (2019). Multi-site evaluation of plastic film mulch and nitrogen fertilization for wheat grain yield, protein content and its components in semiarid areas of China. Field Crops Res..

[77] National Grain and Oil Standardization Technical Committee (1993). Method for Determination of Wet Gluten in Flour. GB/T 14608-1993. https://std.samr.gov.cn/gb/search/gbDetailed?id=71F772D78CA3D3A7E05397BE0A0AB82A.

[78] Ministry of Internal Trade of the PRC (1995). Method for the Determination of Wet Gluten Quality in Wheat Flour-Gluten Index. LS/T 6102-1995. https://std.samr.gov.cn/hb/search/stdHBDetailed?id=8B1827F178E4BB19E05397BE0A0AB44A.

[79] State Administration for Market Regulation (2019). Inspection of Grain and Oils-Doughs Rheological Properties Determination of Wheat Flour-Farinograph Test. GB/T 14614-2019. https://openstd.samr.gov.cn/bzgk/gb/newGbInfo?hcno=9C02083394E746C264A72D8717617CF4.

