# Supplementary material for: Drip Fertigation Enhances the Responses of Grain Yield and Quality to Nitrogen Topdressing Rate in Irrigated Winter Wheat in North China
Source: Plants (Basel). 2024 May 22;13(11):1439. doi: 10.3390/plants13111439 (PMC11174403; doi:10.3390/plants13111439)
Supplement: Supplementary file 1 [file plants-13-01439-s001.zip › plants-2998008-supplementary.pdf]

Table S1. Irrigation amount and nitrogen fertilization rate at different growing stages of winter wheat in the 2021–2022 and 2022–2023 growing seasons.

Growing season	Management practice	N treatment	Irrigation (mm)					Nitrogen fertilizer (kg ha^{-1})		
			WS	JS	BS	FS	Total	PS	JS	Total
2021-2022	FB	N0	60	60	60	60	240	0	0	0
		T0	60	60	60	60	240	150	0	150
		T40	60	60	60	60	240	150	40	190
		T80	60	60	60	60	240	150	80	230
		T120	60	60	60	60	240	150	120	270
		T160	60	60	60	60	240	150	160	310
	DF	N0	60	40	40	40	180	0	0	0
		T0	60	40	40	40	180	150	0	150
		T40	60	40	40	40	180	150	40	190
		T80	60	40	40	40	180	150	80	230
		T120	60	40	40	40	180	150	120	270
		T160	60	40	40	40	180	150	160	310
2022-2023	FB	N0	60	60	60		180	0	0	0
		T0	60	60	60		180	150	0	150
		T40	60	60	60		180	150	40	190
		T80	60	60	60		180	150	80	230
		T120	60	60	60		180	150	120	270
		T160	60	60	60		180	150	160	310
	DF	N0	60	40	40		140	0	0	0
		T0	60	40	40		140	150	0	150
		T40	60	40	40		140	150	40	190
		T80	60	40	40		140	150	80	230
		T120	60	40	40		140	150	120	270
		T160	60	40	40		140	150	160	310

FB, flood irrigation and broadcast fertilizer; DP, drip fertigation; WS, wintering stage; JS, jointing stage; BS, booting stage; FS, filling stage; PS, pre-sowing.

Supplemental figure legend

Figure S1. The CAT and SOD activities, and MDA contents of flag leaves after anthesis in the 2021–2022 growing season. The * and ** indicates there is significant difference between FB and DF according to Student's *t* test at α=0.05 and 0.01, respectively. The blue and red vertical bars represent least significant differences under FB and DF (α=0.05), respectively.

Figure S2. The CAT and SOD activities, and MDA contents of flag leaves after anthesis in the 2022–2023 growing season. The * and ** indicates there is significant difference between FB and DF according to Student's *t* test at α=0.05 and 0.01, respectively. The blue and red vertical bars represent least significant differences under FB and DF (α=0.05), respectively.

Figure S1

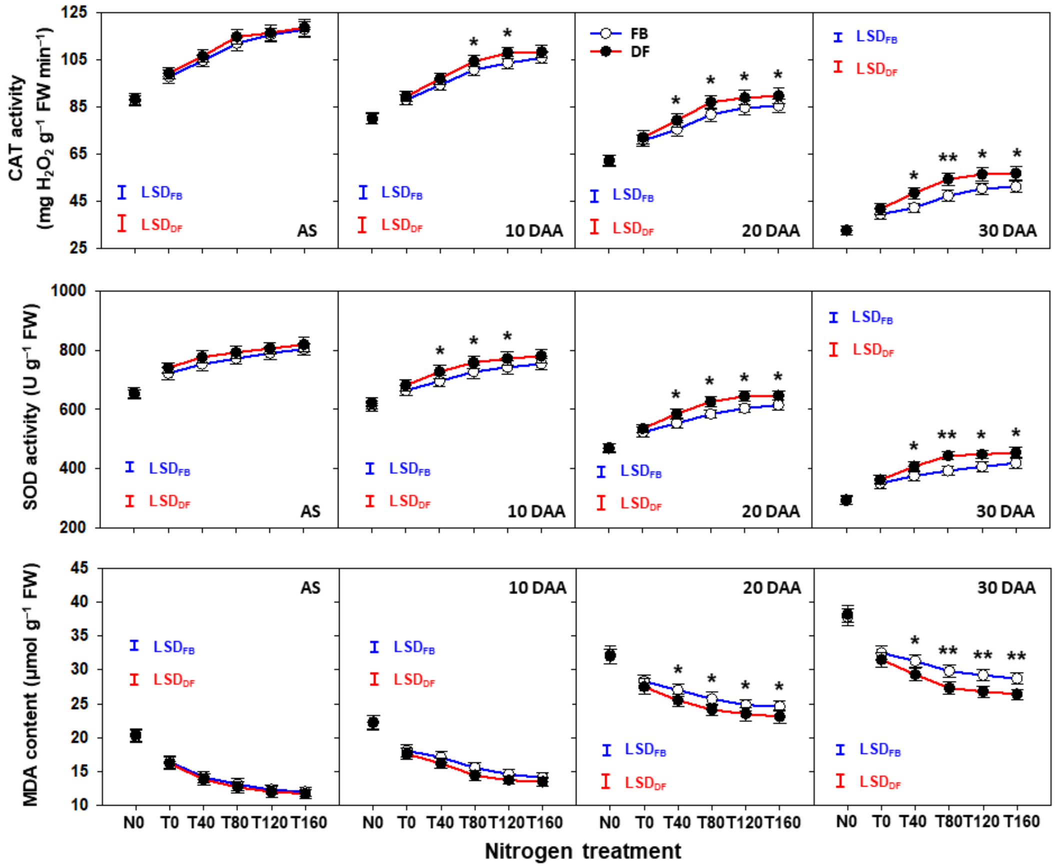

Figure S1. The CAT and SOD activities, and MDA contents of flag leaves after anthesis in the 2021–2022 growing season. The * and ** indicates there is significant difference between FB and DF according to Student's *t* test at α=0.05 and 0.01, respectively. The blue and red vertical bars represent least significant differences under FB and DF (α=0.05), respectively.

Figure S2

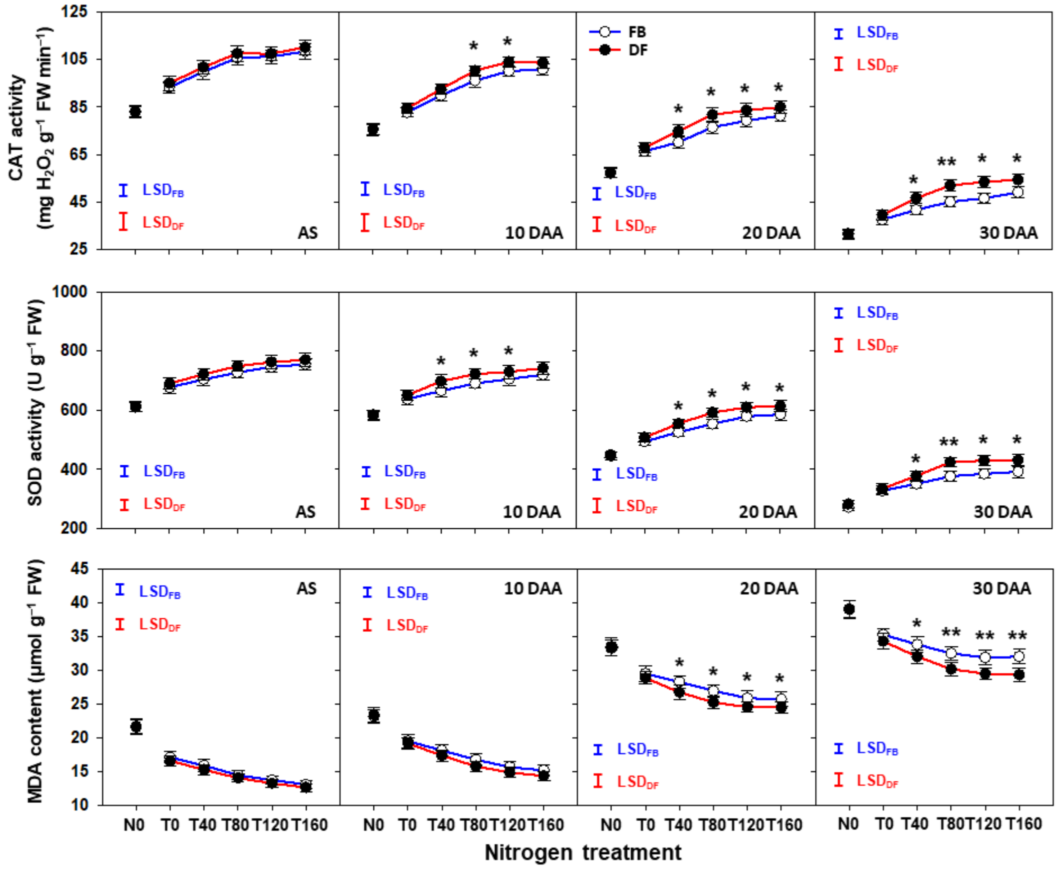

Figure S2. The CAT and SOD activities, and MDA contents of flag leaves after anthesis in the 2022–2023 growing season. The * and ** indicates there is significant difference between FB and DF according to Student's *t* test at α=0.05 and 0.01, respectively. The blue and red vertical bars represent least significant differences under FB and DF (α=0.05), respectively.
